# Deep Learning-Based Automated Segmentation and Quantification of the Ellipsoid Zone and the RPE–Bruch’s Membrane Complex in Healthy Subjects and in Geographic Atrophy

**DOI:** 10.3390/diagnostics16121872

**Published:** 2026-06-16

**Authors:** Nasiq Hasan, Adarsh Gadari, Sharat Chandra Vupparaboina, Elham Sadeghi, Giulia Gregori, Utkarsh Doshi, José-Alain Sahel, Sandeep Chandra Bollepalli, Kiran Kumar Vupparaboina, Jay Chhablani

**Affiliations:** 1 University of Pittsburgh Medical Center, Pittsburgh, PA 15219, USA; nasiq.imtiaz@gmail.com (N.H.); el.sadeghi91@gmail.com (E.S.); ggregori98@gmail.com (G.G.); sahelja@upmc.edu (J.-A.S.); 2 Department of Ophthalmology, University of Pittsburgh, Pittsburgh, PA 15219, USA; adg199@pitt.edu (A.G.); vsharat22@gmail.com (S.C.V.); utd7@pitt.edu (U.D.); bschnd@gmail.com (S.C.B.); kkv@pitt.edu (K.K.V.); 3 Eye Clinic, Department of Experimental and Clinical Medicine, Polytechnic University of Marche, 60121 Ancona, Italy; 4 UPMC Vision Institute, 1622, Locust Street, Pittsburgh, PA 15219, USA

**Keywords:** deep learning, artificial intelligence, ellipsoid zone, RPE–Bruch’s membrane

## Abstract

**Purpose:** This study aimed to validate a deep learning algorithm for automated segmentation and quantitative assessment of the ellipsoid zone (EZ) and the retinal pigment epithelium (RPE)–Bruch’s membrane (BM) complex in healthy eyes and geographic atrophy (GA) eyes. **Methods:** In this retrospective study, spectral-domain optical coherence tomography (SD-OCT) volume scans from 30 healthy eyes and 30 eyes with GA were analyzed. An NMI-Outer Retina Analyzer was used to segment the inner EZ, inner RPE, and outer BM. Average thicknesses of the EZ-RPE, EZ-BM, and RPE-BM were calculated from volumes and across nine ETDRS sectors. Manual segmentations were corrected by two masked expert graders and compared using intraclass correlation coefficients (ICCs). Dice coefficients (DCs), Pearson correlation coefficients, and absolute thickness differences were used to assess agreement between automated and manual segmentation. Heat maps were generated to visualize thickness. **Results:** Thirty healthy eyes and thirty GA eyes were included in the analysis. Mean EZ-RPE, EZ-BM, and RPE-BM thicknesses were 47.55 ± 6.75 µm, 69.49 ± 6.92 µm, and 21.94 ± 3.46 µm, respectively, in the healthy eyes and 15.65 ± 11.09 µm, 39.18 ± 23.28 µm, and 23.52 ± 16.21 µm, respectively, in GA eyes. The model demonstrated high segmentation accuracy, with a mean DC of 0.998 in healthy eyes and 0.995–0.998 in GA eyes. In healthy eyes, differences between automated and manual measurements were minimal (1.42 ± 3.39 μm (2.98%) for EZ-RPE, 1.31 ± 3.18 μm (1.88%) for EZ-BM, and 0.67 ± 1.71 μm (3.05%) for RPE-BM), all within 1.88–3.05% of the gold standard (manual corrections). In contrast, GA eyes showed greater variability (mean differences of 3.61 ± 8.62 μm (23.06%) for EZ-RPE, 4.28 ± 11.34 μm (10.92%) for EZ-BM, and 4.4 ± 10.45 μm (18.71%) for RPE-BM). Heat maps revealed increased variability in the junctional zone surrounding the atrophy. Automated and manual measurements showed strong correlations across all sectors in GA eyes (r = 0.97 for EZ-BM, 0.96 for EZ-RPE, and 0.89 for RPE-BM). **Conclusions:** The NMI-ORA enables accurate, automated segmentation and quantification of outer retinal layers, with performance comparable to that of expert graders.

## 1. Introduction

Age-related macular degeneration (AMD) is the leading cause of vision loss in the elderly, affecting approximately 200 million people worldwide, with prevalence projected to reach 288 million by 2040. Geographic atrophy (GA) represents an advanced stage of AMD and is associated with irreversible vision loss [1,2]. Recently, two intravitreal agents, pegcetacoplan (Syfovre; Apellis Pharmaceuticals, Waltham, MA, USA) and avacincaptad pegol (Izervay; Iveric Bio, Inc., Parsippany, NJ, USA), have received Food and Drug Administration (FDA) approval for GA, targeting disease progression in patients with established atrophy and visual decline [3]. Pivotal trials supporting these approvals relied on fundus autofluorescence (AF) to quantify GA, defining lesions as hypoautofluorescent areas with a minimum size of 250 μm [4]. However, therapeutic options remain unavailable for earlier stages of AMD, when intervention might offer the greatest potential to prevent progression to GA and subsequent vision loss.

GA is characterized by complete loss of the outer retina and retinal pigment epithelium (RPE). Degeneration of the outer retinal layers precedes RPE loss [5]. Increasing attention has also been directed toward the junctional zone surrounding the areas of atrophy. The definition of junction zone remains inconsistent, with a few studies describing it as a 100 µm margin around the atrophy [6], while others define it as areas with outer retinal loss in the presence of intact RPE [7]. These observations highlight the importance of the outer retinal bands, which may be equally relevant as, if not more than, the RPE in understanding the pathophysiology and progression of GA. Among these bands, the ellipsoid zone (EZ) has been the most evaluated, both qualitatively and quantitatively, as a biomarker of outer retinal integrity. The EZ, previously referred to as the inner segment–outer segment (IS/OS) junction, represents the interface of photoreceptor inner and outer segments on OCT. It is one of the four hyperreflective outer retinal bands and is located between the external limiting membrane (ELM) and the interdigitation zone (IZ). Photoreceptors are highly metabolically active cells with a dense concentration of mitochondria within the ellipsoid portion of the inner segment, which corresponds to the EZ. Due to the high reflectivity of these mitochondria, the EZ appears as a distinct hyperreflective band on OCT [8].

With newer drugs in the pipeline, there has been a paradigm shift from AF to OCT and en face OCT as preferred modalities for quantifying photoreceptor and RPE loss in retinal diseases, including AMD [9]. Clinical trials in macular telangiectasia have already adopted EZ loss as a structural endpoint [10,11]. Compared with AF, OCT B-scans and en face images provide superior structural detail of the EZ and RPE. In GA, a comparative study demonstrated good correlation between AF and OCT-based measurements of progression, although baseline RPE loss was overestimated on AF [12]. Another study reported that retinal sensitivity correlated strongly with photoreceptor damage rather than RPE damage, and RPE loss alone was not predictive of functional decline [13].

Artificial intelligence (AI), particularly deep learning (DL), has increasingly been integrated into medical imaging and healthcare applications owing to its ability to improve efficiency, reduce clinician workload, and enable rapid and accurate quantitative assessment [14]. Recent advances have demonstrated the utility of DL in areas including automated image segmentation, explainable AI frameworks, hybrid bioelectronic systems [15], and multimodal image analysis across a range of medical specialties [16]. These approaches have shown promise in enhancing reproducibility, minimizing interobserver variability, and supporting clinical decision-making in both research and clinical settings. In ophthalmic imaging, DL-based segmentation methods have emerged as valuable tools for the objective assessment of retinal structure and disease progression [17].

Despite these advances, accurate identification and segmentation of the outer retinal bands remain challenging, particularly in diseased eyes. Advances in imaging led to the development of automated segmentation models using traditional image processing and advanced deep learning methods [18]. However, the performance of deep learning methods depends on the training data and the quality of manual annotations. Since annotating retinal layers such as the ellipsoid zone (EZ), retinal pigment epithelium (RPE), and Bruch’s membrane is time consuming and can lead to annotator fatigue and reduced annotation quality, the availability of large, high-quality labeled datasets required for training advanced deep learning (DL) models remains limited in practice [19]. Additionally, DL-based segmentation has previously been applied to the EZ and RPE layers, with most studies primarily focusing on quantifying areas of attenuation or loss of these structures [20,21]. While average thickness measurements of these outer retinal layers have been explored in a limited number of disease contexts, such as retinitis pigmentosa [22,23], quantitative assessment of average EZ and RPE thickness parameters remains relatively underexplored in the literature. In light of these challenges, we validate a tool that enables near-accurate segmentation of the EZ and RPE–Bruch’s membrane complex in both healthy and GA eyes, along with quantitative assessment of these layers, with potential applications for future clinical trials and clinical practice.

## 2. Methods

### 2.1. Dataset

This retrospective study was conducted in the Department of Ophthalmology at the University of Pittsburgh Medical Center, in accordance with the Declaration of Helsinki and approved by the Institutional Review Board. OCT volume scans from 30 healthy eyes of participants aged more than 55 years and 30 eyes with conventional AMD-related GA were analyzed for automated segmentation of the EZ and RPE–Bruch’s membrane complex. To avoid inter-eye correlation bias, only one eye per patient was included in the analysis. This retrospective study utilized archived OCT scans obtained from the institutional imaging database. Data were accessed between 15 January 2025 and 30 March 2025. The investigators had access only to limited clinical information, including patient age and diagnosis. No direct identifiers (such as names, medical record numbers, or contact information) were available to the study team during or after data collection.

Spectral-domain OCT volume scans were obtained using the Heidelberg Spectralis system (Heidelberg Engineering, Heidelberg, Germany). Scans with a quality score of less than 20 were excluded. For healthy eyes, a 49-raster protocol was used, while eyes with GA were imaged with a 97-raster protocol. All scans covered a 6 × 6 mm area centered on the fovea, with each B-scan consisting of 512 × 496 pixels. The Early Treatment Diabetic Retinopathy Study (ETDRS) grid was applied to delineate subfields, including the central 1 mm foveal zone, the 3 mm inner ring (superior, temporal, inferior, and nasal), and the 6 mm outer ring (superior, temporal, inferior, and nasal).

### 2.2. Algorithm

Proprietary software developed by NetraMind Innovations, Inc. (Pittsburgh, PA, USA) called the NMI-ORA (Version 1.0) (Outer Retina Analyzer) was used to automatically segment three outer retinal layers: the inner border of the EZ, the inner border of the RPE, and the outer border of Bruch’s membrane (BM). The proposed algorithm employs a two-module framework designed to achieve accurate and biologically consistent layer segmentation. The first module integrates deep learning techniques with pseudo-labeling strategies and traditional image processing methods to delineate retinal layers. The second module, a deep learning model for geographic atrophy (GA) segmentation, detects GA regions and aligns the EZ and RPE boundaries with the BM boundary in accordance with pathological features. Together, these modules operate synergistically to improve segmentation reliability. Algorithm performance was evaluated by quantifying the extent of manual correction required to address segmentation errors, based on the practical observation that adjusting existing boundaries demands considerably less effort than annotating them de novo. Evaluation metrics included mean difference, mean absolute difference, and correlation coefficient, computed for each segmented boundary across A-scan, B-scan, volumetric, and ETDRS-grid levels of analysis—manual tool.

### 2.3. Evaluation

Following automated segmentation, two masked experienced graders (NH and GG) performed manual corrections of retinal layer boundaries where necessary. Three thickness parameters were defined: EZ-RPE thickness (inner ellipsoid zone to inner RPE), EZ-BM thickness (inner ellipsoid zone to outer Bruch’s membrane), and RPE-BM thickness (inner RPE to outer Bruch’s membrane). Intergrader agreement was calculated, and any discrepancies were resolved by consulting the senior author (JC) for the final decision. The outer EZ boundary was excluded from calculations due to the frequently indistinct demarcation between the EZ and IZ in pathological cases, particularly in GA. Thickness measurements were calculated for both automated and manually corrected segmentations to enable comparative analysis.

Average thickness measurements for the EZ-RPE, EZ-BM, and RPE-BM layers were computed across the entire scan volume and within the nine standardized ETDRS subfields. These calculations were performed on both the automated segmentations and the manual corrections. The absolute differences between automated and manual thickness values were subsequently analyzed to quantify segmentation accuracy. Additionally, pixel-wise discrepancies between automated and manual segmentation boundaries were assessed for all three retinal layers.

### 2.4. Statistical Analysis

Statistical analyses were performed using Python version 3.10 (Python Software Foundation, Wilmington, DE, USA) using relevant scientific computing and statistical libraries. Thickness values were expressed as mean ± standard deviation, calculated from individual A-scan measurements. Average volume thicknesses and nine ETDRS subfield thicknesses were measured for all three layers. Interobserver variability was assessed using the intraclass coefficient (ICC). The Dice coefficient (DC) was used to assess agreement between automated and manually corrected segmentations, and the Pearson correlation coefficient was used to assess the relationship between automatically measured and manually corrected thickness values.

## 3. Results

A total of 60 eyes of 60 patients were included in the final analysis. In the healthy subgroup (30 eyes), the mean patient age was 63.11 ± 6.46 years, with 16/30 (53.3%) females. Mean thicknesses were 47.55 ± 6.75 μm for EZ–RPE, 69.49 ± 6.92 μm for EZ–BM, and 21.94 ± 3.46 μm for RPE–BM. In the GA subgroup (30 eyes), the mean age was 72.21 ± 10.41 years, with 21/30 (70%) females. The mean GA area was 8.57 mm^2^, and the total EZ loss area was 13.29 mm^2^. Mean thicknesses were 15.65 ± 11.09 μm (EZ–RPE), 39.18 ± 23.28 μm (EZ–BM), and 23.52 ± 16.21 μm (RPE–BM) (Table 1). Mean EZ–RPE volume was 1.71 ± 0.24 mm^3^ in healthy eyes and 0.56 ± 0.38 mm^3^ in eyes with GA.

**Figure 1:**
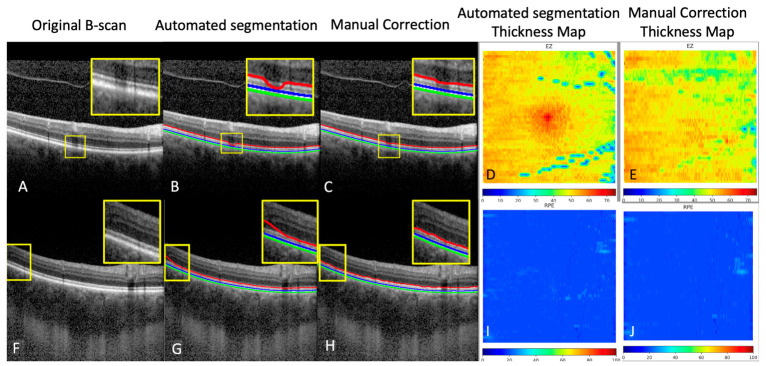
Representative B-scans showing original B-scans (**A**,**F**), automated segmentation (**B**,**G**) and manual corrections (**C**,**H**) of the three boundaries (red—inner boundary of EZ, blue—inner boundary of RPE, green—BM). Yellow boxes indicate areas of segmentation error, beneath retinal vessel shadowing (**B**) and at scan edges (**G**). Corresponding EZ–RPE (**D**,**E**) and RPE–BM (**I**,**J**) thickness heat maps are displayed before and after manual correction. Automated maps demonstrate that most segmentation errors in EZ–RPE thickness occur along the vascular arcades (**D**).

**Figure 2:**
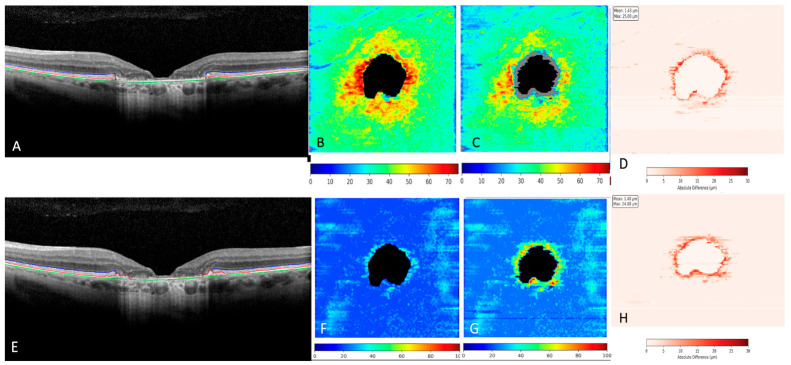
Foveal B-scan demonstrating automated (**A**) versus manually corrected segmentation (**E**) of the of the three boundaries (blue—inner boundary of EZ, red—inner boundary of RPE, green—BM). Corresponding EZ–RPE (**B**,**C**) and RPE–BM thickness (**F**,**G**) heat maps are shown for both automated and manually corrected segmentation. The manually corrected EZ–RPE map highlights (**C**) the area of EZ loss (gray) surrounding the GA. The rightmost heat map displays the absolute differences (**D**,**H**) between automated and manual segmentation respectively, with the greatest corrections observed in the junctional zone around the geographic atrophy.

The segmentation approach performed comparably to a retina expert, with only a small number of scans requiring manual corrections at select points on the B-scan (Figure 1). The ICC between the two manual graders was 0.97 (95% CI: 0.94–0.99). In healthy eyes, the average difference between manual and automated layer thicknesses was 1.42 ± 0.63 (95% CI: 1.18–1.66) μm (2.98%) for EZ–RPE, 1.31 ± 0.56 (95% CI: 1.09–1.52) μm (1.88%) for EZ–BM, and 0.67 ± 0.39 (95% CI: 0.53–0.82) μm (3.05%) for RPE–BM, which is within 1.88–3.05% of the gold standard (manual corrections). Among GA eyes, variability between manual and automated measurements was higher, with mean differences of 3.61+/1.53 (95% CI: 3.02–4.19) μm (23.06%) for EZ–RPE, 4.28 ± 0.86 (95% CI: 3.39–5.18) μm (10.92%) for EZ–BM, and 4.4 ± 2.55 (95% CI: 3.43–5.37) μm (18.71%) for RPE–BM. On examining the heat maps of the difference in absolute thicknesses, we observed that the junctional area around the atrophy contributed to higher differences between the two measurements (Figure 2).

**Figure 3:**
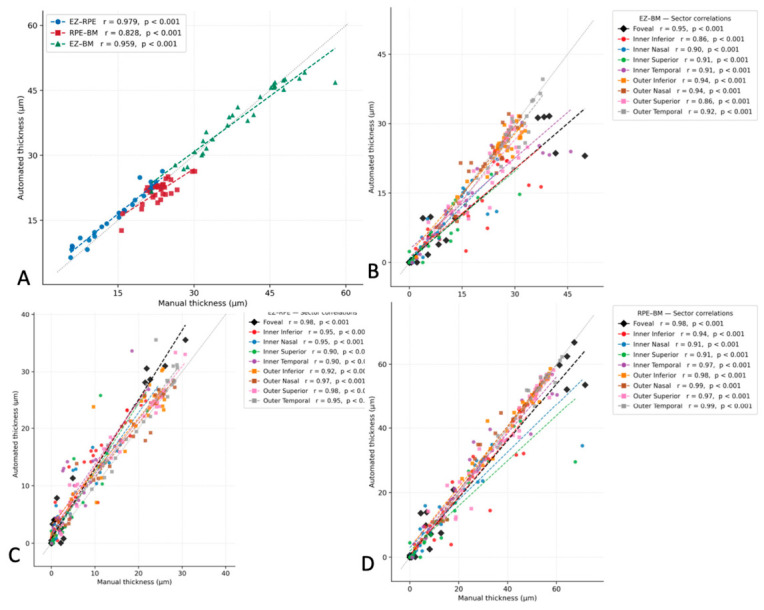
Scatter plots comparing automated versus manual-correction-derived thicknesses of EZ-RPE, RPE-BM and EZ-BM among GA patients (**A**). The dashed lines show the best-fit regression lines for each thickness, with corresponding Pearson correlation coefficients (r) and *p*-values shown in the legend. (**B**–**D**) Scatter plots comparing automated versus manual-correction-derived sectoral thickness measurements of EZ-RPE, RPE-BM and EZ-BM respectively. The algorithm demonstrates strong correlation with manual measurements across all regions, with the highest agreement observed within the fovea.

**Figure 4:**
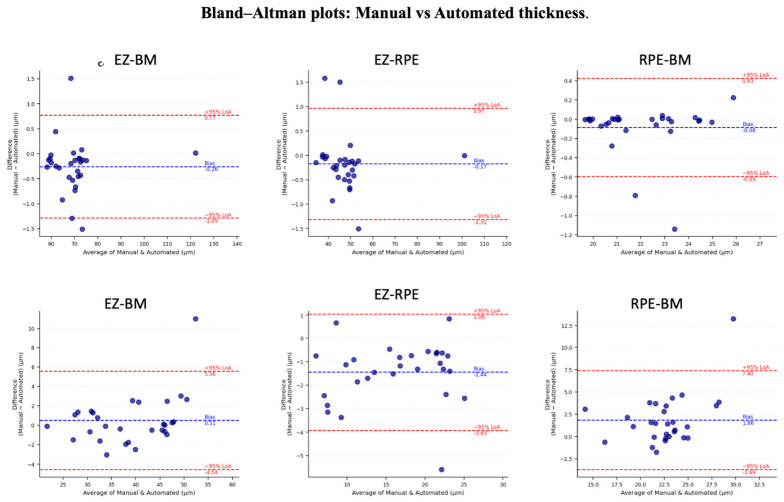
Bland–Altman plots demonstrating agreement between manual and automated measurements for EZ–BM, EZ–RPE, and RPE–BM thicknesses. The upper row represents healthy eyes, while the lower row represents GA eyes. The blue dashed line indicates the mean bias between methods, and the red dashed lines represent the 95% limits of agreement (LoAs). Overall, minimal systematic bias was observed across retinal thickness measurements, with narrower limits of agreement in healthy eyes than in GA eyes.

In the healthy group, the mean DC comparing manual and automated segmentation was 0.998 for all three boundaries. In the GA subgroup, the coefficients ranged from 0.995 to 0.996. Absolute differences in pixels and DC between manual and automated segmentation are summarized in Table 2. When evaluating variability across different ETDRS grid sectors, the average absolute thickness differences ranged from 0.58 to 1.31 μm for EZ–BM, 0.78–1.39 μm for EZ–RPE, and 0.49–0.91 μm for RPE–BM, with higher variability observed in the inner nasal and all outer sectors (Table 3). The mean DC of the boundaries was 0.998 in healthy eyes and ranged from 0.995 to 0.998 in GA eyes (Table 4). Automated and manual measurements demonstrated strong correlations for all outer retinal layer thickness parameters among GA eyes. Correlation was highest for EZ–BM thickness measurements (r = 0.969, *p* = 1.540 × 10^−18^, *n* = 30), followed by EZ–RPE thickness measurements (r = 0.957, *p* = 1.548 × 10^−16^, *n* = 30) and RPE–BM thickness (r = 0.890, *p* = 4.817 × 10^−11^, *n* = 30) (Figure 3). Bland–Altman analysis demonstrated good agreement between automated and manual retinal layer thickness measurements, with relatively small mean biases across EZ–BM, EZ–RPE, and RPE–BM parameters. Agreement was generally stronger in healthy eyes, whereas GA eyes demonstrated wider limits of agreement and greater variability, particularly for EZ-related measurements (Figure 4).

## 4. Discussion

Our study demonstrates a deep learning-based, high-performance automated model for segmentation of the EZ and RPE–BM in both healthy eyes and eyes with AMD-related GA. The results indicate that automated segmentation and thickness measurements closely approximate manual annotations by experienced retinal graders with Dice coefficients of 0.998 in healthy subjects and 0.995–0.998 among GA eyes.

Advances in OCT resolution have allowed the outer retinal layers to be delineated into four distinct bands, and high-resolution OCT imaging has revealed additional sublayers with remarkable clarity [24]. Despite this, visualizing all layers in routine clinical practice and translating these observations into applications for retinal disease remains challenging. Nonetheless, the four primary outer retinal bands remain the standard reference, with the more hyperreflective layers, particularly the RPE–Bruch’s complex and the ellipsoid zone, commonly used for both qualitative and quantitative assessment across various retinal pathologies.

Miranda et al. were among the few to report Dice coefficients for individual retinal layer segmentation, including the ellipsoid zone (EZ). They validated the BioImagingLab/INESC TEC deep learning model against the gold standard of manually corrected automatic segmentation from the Heidelberg Spectralis HRA + OCT software (Version 6.16.8.0) [25]. Dice scores were excellent for most retinal layers in healthy controls (0.928–0.995), particularly the inner retina, but were lower for EZ segmentation (0.783). A similar reduction in EZ accuracy was observed in both intermediate and exudative AMD, despite high performance for other retinal layers and for fluid in exudative AMD. Another group evaluated DL-based retinal layer segmentation of the inner limiting membrane, RPE inner boundary, and Bruch’s membrane using U-Net and DeepLabV3. Their models, trained on Spectralis SD-OCT data and tested on both Spectralis and Cirrus devices, achieved Dice scores of 0.76–0.87, though they did not assess EZ [19]. For patients without AMD, Dice coefficients ranged from 0.83 to 0.84. A study on using the same algorithm in patients with hydroxychloroquine toxicity showed a DICE coefficient of 0.74 ± 0.23 [26]. In comparison, our model achieved superior performance in retinal layer segmentation, including the EZ.

Most DL studies have focused on segmenting EZ and RPE loss and attenuation, showing high accuracy in detecting regions of GA and EZ disruption. This approach mainly emphasizes spatial overlap of EZ and RPE loss areas rather than assessing the precision of thickness quantification, leaving a gap in the literature regarding the validation of thickness measurements using Dice metrics. Using absolute EZ thicknesses may enhance clinical trials by providing quantitative measures rather than classifying regions simply as partial or complete attenuation. Such measurements allow evaluation of different levels of EZ risk, and future studies could define thickness thresholds around the GA penumbra associated with mild, moderate, or high risk.

Recent studies have utilized ellipsoid zone morphology to monitor geographic atrophy progression and to evaluate the efficacy of drugs aimed at slowing or halting disease progression. These metrics have been incorporated as clinical trial endpoints in recent studies, including ReCLAIM-2 [27] and FILLY [28]. Orlando et al. reported a Dice coefficient of 0.912 ± 0.021 for the central subfield and 0.907 ± 0.028 for the full sample when comparing automated segmentation to manual grading of the photoreceptor layer (EZ-RPE) in diabetic retinopathy eyes [29]. Wang et al. evaluated an EZ loss detection algorithm based on thresholding of thickness maps derived from layer segmentation, reporting Dice coefficients of 0.88 ± 0.07 for B-scan-based segmentation and 0.63 ± 0.25 for EZ loss detection, as assessed against manual grading of B-scans and ratio images [30]. Similarly, a study by Zhang et al. established a model that automatically classified atrophy as cRORA or iRORA based on RPE loss, photoreceptor degeneration, and hypertransmission, demonstrating excellent performance that exceeded human-to-human consensus [31]. A recent study by Kalra et al. also reported excellent accuracy, with 99% detection of the EZ at risk and 90% measurement accuracy [32]. However, the analysis of these studies focused on the area of atrophy or EZ at risk rather than direct thickness measurements of the retinal layers.

Automated and manual segmentation showed greater variability in GA patients than in healthy controls, particularly within the junctional zone, where differences of up to 25 μm were observed on heat maps. This variability may reflect deposits or abrupt changes in outer retinal morphology. Since the junctional zone is a key area of interest in GA trials, analysis of the EZ and other outer retinal layers in it is especially important. Although the absolute variability was small, the relative percentage shift appeared significant. Minimal manual correction at these points could improve accuracy. We did not assess average thickness across different AMD sectors due to heterogeneity in GA location within our cohort. Future studies should examine junctional zone thickness in greater detail, ideally at set distances (e.g., 100, 200, and 300 µm from the GA margin) and compare these with the normal EZ thickness in the scan periphery.

One of the key advantages of our tool is that it enables manual correction, allowing human graders to verify the accuracy of automated segmentation and perform manual corrections when needed. The difference between automated and manually corrected thickness measurements was minimal (1.88–3.05%). Although there are no universally established thresholds defining clinically acceptable segmentation variability for these measurements, the observed differences corresponded to only a few microns in absolute thickness values. Importantly, these differences were smaller than the axial resolution of the Spectralis SD-OCT system, raising the possibility that some of the observed variations may not represent a true anatomical difference. Therefore, such minimal variability is unlikely to substantially affect overall retinal thickness assessment or clinical interpretation. Moreover, the time required for manual correction is substantially reduced, from several hours to less than 15 min, even in poor-quality OCT volumes. Our model also enables the generation of maps that allow independent measurement of EZ and RPE attenuation and loss and can be further stratified into different levels of risk, which can serve as biomarkers in major clinical trials. The associated heat maps provide a qualitative assessment of GA progression, offering an alternative to reliance on fundus autofluorescence. By comparing EZ and RPE heat maps, areas of the retina at risk can be identified both qualitatively and quantitatively, facilitating evaluation of current and emerging interventions for AMD and GA. This algorithm is readily applicable in both clinical trial settings and routine clinical practice.

Limitations of this study include training and validation of the model using a single OCT device, albeit one that is widely available and commonly used in clinical trials. The sample size was considerably small; however, the algorithm was trained and tested with an external validation dataset. The very high DC observed in this study should be interpreted with caution, as factors such as dataset characteristics and limited heterogeneity may contribute to overestimation of model performance. Although no evidence of data leakage was identified, external validation using larger and more-diverse datasets is necessary to further establish the robustness and generalizability of the algorithm. Manual evaluation of OCT scans at this level requires expert graders and is inherently labor-intensive, with potential for intergrader variability and measurement bias. Another potential limitation of this study is the possibility of anchoring bias, as manual corrections were performed on automated segmentations rather than through fully de novo manual segmentation; however, fully de novo segmentation was considered impractical due to the substantial time and resource requirements associated with large-scale manual annotation. The findings of this study should be interpreted primarily as reflecting human-in-the-loop segmentation performance and reduction in manual edit burden rather than absolute standalone algorithmic accuracy derived from fully independent de novo manual segmentation. These limitations hinder the feasibility of using such assessments in routine clinical practice or as efficacy endpoints in clinical studies. A fully automated three-dimensional quantitative grading system, however, can substantially reduce the time required to analyze each volume.

## 5. Conclusions

We present a novel, fully automated deep learning method for the detection, quantification, and classification of geographic atrophy from OCT scans. The model demonstrated predictive performance comparable to that of clinical experts. This approach has the potential to support patient management and to standardize clinical trial endpoints, thereby facilitating the development of new therapies for GA.

## Figures and Tables

**Table 1:** Mean thickness values following automated segmentation and manual correction and absolute difference in thickness values between automated and manual segmentations of the three layers in healthy and GA subjects.

Group	Layer	Manual Correction (Microns, 95% CI)	Automated Measurement (Microns, 95% CI)	Thickness Difference (Microns, 95% CI)
Healthy	EZ-BM	69.49 ±6.92 (65.17–73.8)	69.74 ± 5.84 (65.43–74.05)	1.31 ± 0.56 (1.09–1.52)
	EZ-RPE	47.55 ± 6.75 (43.16–51.94)	47.72 ± 5.68 (43.31–52.13)	1.42 ± 0.63 (1.18–1.66)
	RPE-BM	21.94 ± 3.46 (21.26–22.61)	22.02 ± 3.57 (21.34–22.69)	0.67 ± 0.39 (0.53–0.82)
GA	EZ-BM	39.18 ± 8.87 (35.81–42.55	38.67 ± 7.93 (35.65–41.69	4.28 ± 0.86 (3.39–5.18)
	EZ-RPE	15.65 ± 6.22 (13.28–18.02)	17.09 ± 6.07 (14.78–19.39)	3.61 ± 1.53 (3.02–4.19)
	RPE-BM	23.52 ± 3.99 (22.01–25.04)	21.66 ± 2.88 (20.57–22.76)	4.4 ± 2.55 (3.43–5.37)

EZ—ellipsoid zone, RPE—retinal pigment epithelium, BM—Bruch’s membrane, CI—confidence interval.

**Table 2:** Mean absolute difference in pixels and Dice coefficient between automated segmentation and manual correction of the three layers in healthy and GA subjects.

Group	Boundary	Absolute Difference (Pixels)	Dice Coefficient
Healthy	EZ	0.249 ± 0.14 (0.19–0.31)	0.998 ± 0.007 (0.997–0.999)
	RPE	0.172 ± 0.096 (0.13–0.21)	0.998 ± 0.006 (0.997–0.999)
	BM	0.142 ± 0.05 (0.12–0.16)	0.998 ± 0.001 (0.998–0.999)
GA	EZ	1.25 ± 3.51 (0.91–1.59)	0.995 ± 0.013 (0.992–0.998)
	RPE	1.33 ± 3.51 (0.99–1.67)	0.995 ± 0.014 (0.992–0.998)
	BM	0.71 ± 4.24 (0.29–1.12)	0.996 ± 0.013 (0.993–0.999)

EZ—ellipsoid zone, RPE—retinal pigment epithelium, BM—Bruch’s membrane, GA—geographic atrophy.

**Table 3:** Mean thickness values of automated segmentation, manual correction and absolute difference in thickness between automated and manual thickness measurements of the three layers within the nine ETDRS sectors in healthy subjects.

Layer	EZ-BM			EZ-RPE			RPE-BM		
Sector	Manual Measurement (Microns)	Automated Measurement (Microns)	Absolute Difference (Microns)	Manual measurement (Microns)	Automated Measurement (Microns)	Absolute Difference (Microns)	Manual measurement (Microns)	Automated Measurement (Microns)	Absolute Difference (Microns)
Foveal	77.34 ± 5.21	77.26 ± 4.94	0.69 ± 1.59	52.94 ± 5.88	52.73 ± 5.46	0.85 ± 1.90	24.40 ± 2.81	24.53 ± 2.82	0.56 ± 1.35
Inner inferior	72.73 ± 4.53	72.58 ± 4.31	0.79 ± 1.75	49.34 ± 4.81	49.07 ± 4.62	0.94 ± 2.02	23.39 ± 2.99	23.51 ± 3.16	0.59 ± 1.42
Inner nasal	73.71 ± 4.61	73.26 ± 4.42	1.04 ± 1.86	50.28 ± 4.86	49.70 ± 4.71	1.20 ± 2.11	23.43 ± 2.81	23.57 ± 3.03	0.67 ± 1.53
Inner superior	72.50 ± 3.73	72.42 ± 3.62	0.58 ± 1.43	49.11 ± 4.26	48.84 ± 4.31	0.78 ± 1.77	23.39 ± 2.89	23.58 ± 3.10	0.60 ± 1.48
Inner temporal	72.46 ± 4.69	72.46 ± 4.51	0.69 ± 1.59	47.96 ± 5.28	47.77 ± 4.95	0.92 ± 2.00	24.50 ± 3.36	24.69 ± 3.45	0.78 ± 1.70
Outer inferior	69.47 ± 5.39	69.65 ± 4.62	1.31 ± 2.78	48.24 ± 5.37	48.43 ± 4.56	1.35 ± 2.85	21.23 ± 2.36	21.22 ± 2.33	0.49 ± 1.26
Outer nasal	69.39 ± 5.31	69.23 ± 4.85	1.29 ± 2.36	47.96 ± 5.26	47.72 ± 4.86	1.39 ± 2.57	21.43 ± 2.45	21.51 ± 2.68	0.61 ± 1.51
Outer superior	69.40 ± 4.80	69.65 ± 4.01	0.94 ± 2.27	47.88 ± 5.06	48.07 ± 4.34	1.02 ± 2.34	21.53 ± 2.48	21.59 ± 2.56	0.52 ± 1.31
Outer temporal	69.62 ± 5.13	69.76 ± 4.87	1.06 ± 2.21	47.14 ± 5.20	47.12 ± 4.75	1.24 ± 2.59	22.48 ± 3.04	22.64 ± 3.19	0.91 ± 1.91

EZ—ellipsoid zone, RPE—retinal pigment epithelium, BM—Bruch’s membrane.

**Table 4:** Dice coefficients between automated segmentation and manual correction of the three boundaries within the nine ETDRS sectors in healthy and GA patients.

ETDRS Sector	Healthy			GA		
	EZ	RPE	BM	EZ	RPE	BM
Foveal	0.999 ± 0.001	0.999 ± 0.001	0.999 ± 0.001	0.996 ± 0.008	0.996 ± 0.007	0.996 ± 0.002
Inner inferior	0.999 ± 0.001	0.999 ± 0.001	0.999 ± 0.001	0.997 ± 0.008	0.996 ± 0.007	0.998 ± 0.002
Inner nasal	0.999 ± 0.001	0.999 ± 0.001	0.999 ± 0.001	0.997 ± 0.011	0.997 ± 0.009	0.998 ± 0.004
Inner superior	0.999 ± 0.001	0.999 ± 0.001	0.999 ± 0.001	0.996 ± 0.007	0.996 ± 0.007	0.997 ± 0.004
Inner temporal	0.999 ± 0.001	0.999 ± 0.001	0.999 ± 0.001	0.995 ± 0.007	0.995 ± 0.006	0.997 ± 0.002
Outer inferior	0.999 ± 0.001	0.999 ± 0.001	0.999 ± 0.001	0.997 ± 0.008	0.997 ± 0.007	0.998 ± 0.003
Outer nasal	0.999 ± 0.001	0.999 ± 0.001	0.999 ± 0.001	0.997 ± 0.011	0.997 ± 0.008	0.998 ± 0.005
Outer superior	0.999 ± 0.001	0.999 ± 0.001	0.999 ± 0.001	0.996 ± 0.008	0.995 ± 0.008	0.997 ± 0.005
Outer temporal	0.999 ± 0.001	0.999 ± 0.001	0.999 ± 0.001	0.996 ± 0.009	0.997 ± 0.008	0.998 ± 0.003

EZ—ellipsoid zone, RPE—retinal pigment epithelium, BM—Bruch’s membrane.

## Data Availability

The data that support the findings of this study are available from the corresponding author upon reasonable request. Due to patient privacy and institutional regulations, the data are not publicly available.
